# Evaluating the Effectiveness of the Nudge Theory in Improving the Oral Self-Care of Schoolchildren with Refugee and Immigrant Backgrounds in Mashhad, Iran

**DOI:** 10.3390/dj12070228

**Published:** 2024-07-19

**Authors:** Seyed Ahmad Banihashem Rad, Marcella Esteves-Oliveira, Ali Kazemian, Negar Azami, Mehrzad Khorshid, Aylin Sohrabi, Amir Attaran Khorasani, Guglielmo Campus

**Affiliations:** 1 Department of Restorative, Preventive and Pediatric Dentistry, University of Bern, Freiburgstrasse 7, 3010 Bern, Switzerland; seyedahmad.banihashemrad@unibe.ch (S.A.B.R.); marcella.esteves@med.uni-tuebingen.de (M.E.-O.); 2 Graduate School for Health Sciences, University of Bern, Mittelstrasse 43, 3012 Bern, Switzerland; 3 Department of Restorative Dentistry and Endodontology, Justus-Liebig-University Giessen, 35390 Giessen, Germany; 4 Department of Conservative Dentistry, Periodontology and Endodontology, University Centre of Dentistry, Oral Medicine and Maxillofacial Surgery (UZMK), University of Tübingen, 72076 Tübingen, Germany; 5 Department of Community Oral Health, School of Dentistry, Mashhad University of Medical Sciences, Mashhad 9177948959, Iran; kazemiana@mums.ac.ir; 6 Department of Dentistry, Mashhad University of Medical Sciences, Mashhad 9177948959, Iran; negar.azami.info@gmail.com (N.A.); mehrzad.khorshid@gmail.com (M.K.); ailin.sohraby@gmail.com (A.S.); attaranka971@mums.ac.ir (A.A.K.); 7 Department of Cariology, Saveetha Dental College and Hospitals, SIMATS DEEMED University, Chennai 600077, India; 8 Department of Oral and Maxillofacial Sciences, Sapienza University of Rome, Piazzale Aldo Moro 5, 00185 Rome, Italy

**Keywords:** oral health education, Nudge theory, plaque index, intervention, DMFT, dental caries, dental public health, pediatric dentistry, Iran

## Abstract

Nudge theory proposes using subtle interventions to encourage individuals to make better decisions. The aim of this study was to evaluate the effectiveness of the Nudge theory in plaque control and assess caries experience among third-grade primary schoolchildren with refugee and immigrant backgrounds in Mashhad, Iran. Moreover, Afghan and Iranian schoolchildren were compared to assess differences in oral health outcomes. A quasi-experimental field trial was conducted in three public primary schools, comprising 309 participants approximately 9 years old. Interventions were randomly assigned to three schools: School I Messages based on Social Norms (MSN), School II Messages based on Fear of Negative Outcome (MFNO), and School III control group (C). MSN and MFNO received customized motivational video clips at baseline, while C only received Oral hygiene instruction (OHI). All participants received OHI, a brush, and toothpaste. Baseline plaque index (PI) and caries experience in primary and permanent dentition (dmft/DMFT) were recorded. PI was reassessed at two weeks, two months, and six months post-intervention. All data were subjected to statistical analysis. The mean PI decreased significantly in all three groups at the two-week follow-up (*p* < 0.01). The PI improvements declined over a six-month follow-up period in all groups, and the mean PI difference after six months compared to the pre-intervention was significant only in MSN and MFNO (*p* < 0.01), while C reverted almost to the pre-study level. Schoolchildren with at least one filled tooth or Iranian nationality showed a greater PI reduction (*p* < 0.01, *p* = 0.05). The overall mean ± SD dmft and DMFT were 4.24 ± 2.11 and 1.70 ± 1.24, respectively. Among all the examined participants, 32 (10.40%) individuals were caries-free. The mean dmft was statistically significantly higher in Afghan children than in Iranians (*p* = 0.01). MSN was more effective on PI reduction in the short term, while MFNO was more long-lasting. Using the Nudge theory via visual aids was more effective in motivating children to perform better oral self-care than solely traditional OHI.

## 1. Introduction

According to the United Nations High Commissioner for Refugees (UNHCR), the worldwide refugee population reached 100 million in 2022 [1,2]. Simultaneously, the global population of immigrants reached 281 million in 2020, as stated in the World Migration Report [3]. Despite the increasing number of refugees and immigrants globally, there is limited research on oral health-promotion interventions within this population [4].

The global prevalence of oral health diseases (i.e., dental caries and periodontal problems) is a constant reminder of the global need for appropriate oral health education and prevention programmes [5,6]. Oral health education is an essential component of the acquisition of healthy behaviors [7]. A meta-analysis [8] supported all oral health education interventions, such as improving individuals’ knowledge, self-care and health behaviors, including brushing and flossing.

Previous studies have highlighted the high prevalence of oral health problems among refugees and the importance of providing oral health care to this population [9]. Moreover, dental diseases in Western cultures might profoundly impact self-image, self-esteem, social behaviors, employability, housing and social perceptions by others [10]. Therefore, interventions aimed at enhancing health literacy should focus on disadvantaged and migrant populations [11,12]. Ultimately, the assimilation of immigrants and refugees might be facilitated by improvement in their oral health.

Proper oral health is essential to a person’s overall health and quality of life [13]. An individual’s oral health includes being able to properly speak, smile, taste, chew, swallow and express a variety of emotions without experiencing pain or discomfort [14]. Some aspects of health can be improved by simple behavioral changes, such as oral hygiene.

The findings of behavioral economics, a new branch of economics that incorporates findings from psychology into economics, could be useful in promoting healthy behaviors. Two Nobel Prizes in Economics were awarded in 2002 and 2017 to Daniel Kahneman and Richard Thaler, respectively, who brought behavioral economics to the attention of various academic disciplines.

Thaler’s theory, known as the Nudge theory, deals with cheap and easy interventions that are effective in changing people’s behavior. According to Nudge theory, desirable and healthy behaviors can be encouraged through Easy, Attractive, Social and Timely interventions (EAST) [15]. The Nudge theory was originally described for behavioral economics, but it applies to several fields of science, as it mainly describes ways to influence decision-making without coercion. A nudge makes it more likely that an individual will make a particular choice, or behave in a particular way, by altering the environment so that automatic cognitive processes are triggered to favor the desired outcome [16].

In the context of oral health, Nudge theory has been applied in different ways to affect people’s behavior. Dental practices could employ choice architecture by offering discounts for positive oral health behaviors during check-ups to incentivize patients for better oral health maintenance. Additionally, sending personalized text message reminders to patients to schedule dental check-ups proved to improve consistent oral hygiene [17,18]. Interventions based on social norms, such as campaigns highlighting the prevalence of good oral health habits among peers, encourage similar behaviors [19]. Visual cues such as posters and pamphlets could potentially reinforce healthy oral hygiene practices. Behavioral economics principles are also utilized by framing dental treatments in terms of long-term health benefits to address patient fears to encourage patients to seek out dental treatment in a timely and proactive manner. These applications demonstrate how targeted nudges can effectively promote positive oral health behaviors and decisions [17,20]

Iran is home to one of the largest and most protracted urban refugee populations in the world, according to UNHCR. Iran has a high number of Afghan migrants and refugees [21] who have been fleeing their homeland for decades because of war, insecurity, violence, drought and unemployment. More than 4.5 million Afghans live under a variety of legal, economic and social conditions in Iran [22]. Mashhad is an Iranian city in the north-eastern part of the Iranian plateau. It is the second largest city, with the highest number of foreign immigrants after Tehran and with some suburbs comprised primarily of Afghan immigrants [23]. Despite the widespread presence of Afghans in Iran, their oral health status has rarely been studied.

Social norms play an important role in influencing behavior by showcasing what others do in similar situations, while the fear of negative outcomes emphasizes the negative consequences of adopting a behavior [24,25,26]. These concepts were applied by showing video clips to schoolchildren suggesting that proper oral self-care is a social norm nowadays in one intervention group and indicating the possible negative consequences of not adopting proper oral self-care in another intervention group.

Given the advantages of prevention over treatment and the cultural weakness of the general public regarding oral health, as well as the high cost of treatment needed by society and the attention of international organizations to prevention, the importance of prevention methods and research in this regard is clear.

There is little existing literature about the Nudge implications in oral health [27,28]; thus, a field trial was conducted to evaluate the effectiveness of the Nudge theory in the promotion of better oral self-care and behavioral change of third-grade primary schoolchildren with refugee and immigrant background in Mashhad, Iran. Moreover, the study aimed to assess differences in oral health outcomes, caries prevalence and changes in plaque index (PI) among Afghan and Iranian schoolchildren following the interventions. Additionally, the relationship between age and nationality with caries experience was evaluated. A better understanding of behavioral Nudges might assist policymakers, clinicians and researchers in developing and implementing useful nudge interventions to improve oral health.

## 2. Materials and Methods

### 2.1. Trial Design and Study Participants

The study was based on the pattern of quasi-experimental studies as a population- intervention with non-random allocation. The field trial was conducted in three public primary schools with immigrants and refugee backgrounds (similar in socioeconomic status and standard of teaching) in the fifth district of Mashhad.

### 2.2. Ethical Considerations and Trial Registration

The field trial protocol was registered and approved by the Ethical Committee at Mashhad Dental School under the reference number IR.MUMS.DENTISTRY.REC.1398.002. A one-time verbal consent was obtained from each subject in the presence of the class teacher.

### 2.3. Sample Size

Sample size calculation was based on the plaque index (PI) (effect size (ES) = 0.28, α err prob = 0.05, Power (1−β err prob) = 0.99), and the total sample size was estimated to be 256. F test ANOVA via G*Power 3.1 was used to calculate the sample size [29,30]. Considering the possibility of attrition, the sample size was increased.

### 2.4. Eligibility Criteria

The inclusion criteria required that children were in good general health and enrolled in the third grade (approximately 9 years of age), and had supplied the written consent form signed by parents/guardians. This study excluded children who were taking medication known to reduce the salivary flow rate, as well as those who had systemic conditions or chronic diseases. Exclusions were also made for children who were absent on examination days or refused to participate.

### 2.5. Randomization

Three public primary schools were randomly selected by a random sequence generator out of the boys’ public school list of the fifth district of Mashhad using the RAND function to generate random numbers for sorting, with the top three schools being chosen for the study [31]. All third-grade students in all three schools were enrolled in the study by the census method. The question of which school receives which intervention was decided by a random sequence generator in Microsoft Excel (Version 16.55).

### 2.6. Blinding

The examiners were blinded to the study groups and types of interventions. A coding scheme of School I Messages based on Social Norms (MSN), School II Messages based on Fear of Negative Outcome (MFNO) and School III control group (C) was used for the different groups. This coding scheme was not revealed during data entry and analysis.

### 2.7. Questionnaire and Content Validity

A pilot study was performed on 32 children aged 9 and 10 years to check the comprehension of the video clips. These subjects were not enrolled in the study.

Two types of questionnaires were designed for MSN and MFNO groups. Each questionnaire consisted of 10 questions, of which three items were relevant questions (Likert-scale questions) and seven items were binary questions (yes/no questions). The content of the structured questionnaires was validated by 10 subject experts. The Likert-scale for the relevant questions was as follows: (1) not relevant, (2) somewhat relevant, (3) quite relevant and (4) highly relevant. The number of experts in agreement (scored 3 or 4) for each item was calculated. The questionnaires used for the content validity of video clips were already validated [32,33].

### 2.8. Calibration of Examiners

Three examiners were calibrated before the clinical examinations, for measuring the dmft/DMFT and PI on 10 children with similar age under supervision of an Associate professor of Mashhad Dental School acting as a benchmark. The inter-rater agreements among examiners for each item, decayed, missing and filled teeth (for dmft/DMFT index) and plaque score (at tooth surface level for PI), were measured by Fleiss’ Kappa.

### 2.9. Clinical Examination

Each participant underwent a comprehensive examination in the classroom or school hall, where they were seated in reclining chairs. In accordance with the World Health Organization’s recommended protocols, portable lights, dental mirrors, gloves, tongue blades and periodontal probe were used in a standardized environment during the examination process [34]. During the baseline examination, the PI was initially recorded, followed by the assessment of dmft/DMFT, which was facilitated by the use of cotton rolls to ensure a dry oral environment. Further moisture mitigation was achieved by applying cotton rolls and gauze.

The plaque index (PI) of the Silness–Loe Plaque Index was recorded. These six teeth were selected to shorten the examination time. The Silness–Loe [35] PI includes the following teeth:

1. Maxillary Right First Molar (Tooth #16);
2. Maxillary Right Lateral Incisor (Tooth #12);
3. Maxillary Left First Bicuspid (Tooth #24);
4. Mandibular Left First Molar (Tooth #36);
5. Mandibular Left Lateral Incisor (Tooth #32);
6. Mandibular Right First Bicuspid (Tooth #44).

To record the PI, the plaque present in the cervical edge of the distobuccal, buccal, mesiobuccal and lingual (four) surfaces of each tooth was evaluated using a probe in appropriate light. Each level was given a score between 0 and 3 based on the amount of plaque present, and at the end, the PI of an individual was determined by summing the values obtained for each tooth and calculating the averages.

In cases where any of the selected teeth were missing, no replacement was provided, and the index was calculated based on all the existing teeth. The scoring scheme for the PI is included below:

0: Absence of plaque;

1: When the plaque is not visible to the naked eye and can be detected only by a probe;

2: Average accumulation of plaque that is visible to the eye;

3: The large volume of debris and plaque.

### 2.10. Intervention

The study was conducted between April and October 2023. The examinations were conducted between 9 and 11 in the morning at schools.

The field trial focused on providing oral health messages to children. The principal investigator (SABR) was always present in the students’ classes to provide the health messages/intervention.

In the present study, interventions were designed to improve oral self-care in children with refugee and immigrant backgrounds using two messages from Nudge theory: the effect of social norms, and the fear of negative outcome.

The population enrolled was randomly divided into three groups on school-based:

Case group (MSN): Participants watched a video featuring interviews of children who had good oral health and expressions of satisfaction with their hygiene habits. The speech that accompanied it emphasized the prevalence of good oral hygiene among peers of comparable age. This intervention’s main goal was to give participants the impression that maintaining good oral hygiene, which includes brushing twice a day and limiting the intake frequency of sugary snacks, is a social norm that is widely recognized by their peers. For this purpose, interviews with peers who supported these activities as the normative standard were shown to the participants. In addition, this group received the control group’s intervention.

Case group (MFNO): Participants watched a video featuring interviews of children who had poor oral health and complained about oral health issues due to poor oral hygiene, talking about their experiences of pain and discomfort. The speech that accompanied it emphasized the potential negative consequences of poor oral hygiene practices and high sugar-intake frequency. This intervention’s main goal was to instil fear-based messages about the negative consequences of poor oral self-care, emphasizing the importance of regular oral hygiene practices to prevent similar negative outcomes. In addition, this group received the control group’s intervention.

Control group (C): The control group received adequate tools and skills in tooth brushing, flossing and tongue cleaning (frequency, duration and technique). The proper technique for brushing [36] and flossing was demonstrated to students via video and on a dentiform.

All students received an oral health package containing a brush and toothpaste in the first examination. In a two-week follow-up, students received a leaflet designed based on the relative intervention group as a reminder (Second intervention). On the back of the leaflet, a calendar was drawn to encourage students to keep the leaflet. Despite the fact that the interventions and leaflets were primarily in Farsi, a translated version is attached in the Supplementary Materials (Figures S1, S2 and S3 on pages 2, 3 and 4).

The content of video clips in case groups included responses to the following interview questions:

1. Do you brush your teeth? How many times a day and when?
2. How much do you like/eat chocolate and sweets?
3. Have you ever had a toothache? When did you feel the pain? What did you do about it?
4. Have you ever been to a dentist? What did they do for you?

### 2.11. Evaluation of Intervention

The Plaque Index (PI) [35], which has a score between 0 and 3, was assessed a total of four times (before the intervention, and two weeks, two months and six months [37] after the first examination) to measure the effectiveness of behavior-change methods. The PI was recorded by three calibrated dentists.

### 2.12. Data Analysis

The content validity of the two questionnaires was evaluated by means of the Content Validity Index (CVI), which was calculated for each item (question) and both questionnaires using Microsoft Excel. The Item-Content Validity Index (I-CVI) for each question, as well as the Scale-Content Validity Index (S-CVI), which is the overall scale (the average of I-CVI scores) for each questionnaire, were measured. Kappa–Cohen (K*) was used to determine the inter-rater agreement among the experts. After the trial was completed, the data obtained were entered into Microsoft Excel 2016 (Microsoft Corporation, Washington, DC, USA) and analyzed using the IBM SPSS Statistics (Version 27). Tests such as the chi-square test for categorical data and parametric tests such as Repeated Measures ANOVA (Analysis of Variance), Independent *t*-test and One-Way ANOVA for quantitative data were used, and *p*-value less than 0.05 was considered statistically significant. The Chi-square test was applied to evaluate the significance of study characteristics on a categorical scale and measure the percentage of caries-free individuals. 

Repeated Measures ANOVA test was used to determine whether there were differences in PI changes among participants across the three groups. When the ANOVA test showed significant differences, post hoc Tukey’s test identified which means differed. The relationship between dmft/DMFT and age was measured using One-Way Analysis of Variance (ANOVA), while the relationship with nationality was measured using the Independent Samples *t*-test.

The assumptions of parametric tests were checked using the Shapiro–Wilk test (*p <* 0.05). Parametric tests, such as ANOVA, were used because they are robust to violations of normality with large sample sizes (N: 309) [38,39,40]. Bonferroni correction was used for pairwise and multiple comparisons.

## 3. Results

### 3.1. Study Sample

**Figure 1:**
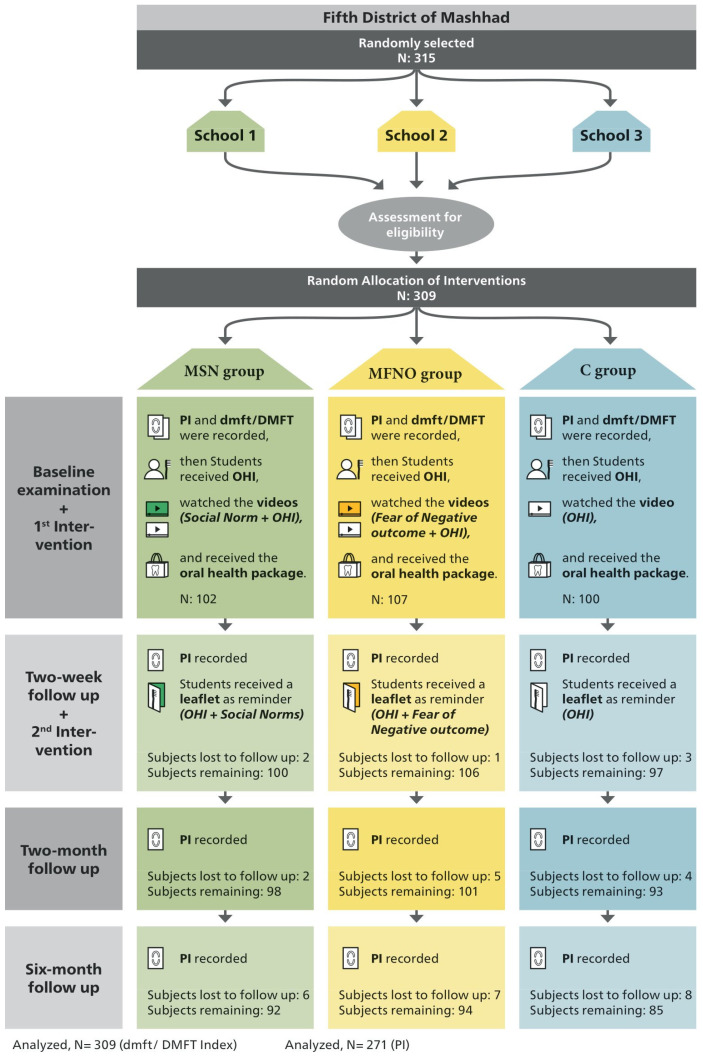
Schematic representation of study procedure. N: Number; MSN: Messages based on “Social Norms”; MFNO: Messages based on “Fear of Negative Outcome”; C: Control; OHI: Oral Hygiene Instruction; PI: Plaque Index; DMFT: Caries experience in the permanent dentition; dmft: Caries experience in the primary dentition.

Three public primary schools were randomly selected from boys’ public schools (totaling 16 schools) in the fifth district of Mashhad. Using a census approach, a total of 315 students were enrolled. Upon fulfilling the inclusion criteria, 309 students were included in the study. There were 102, 107 and 100 students from the MSN group (Kashani School), MFNO group (Imam Sadegh School) and control group (Pasdaran School), respectively. The schoolchildren underwent a baseline examination to determine PI and dmft/DMFT indices. In total, 38 individuals were lost to follow-up due to absence on the day of the examination. Consequently, the PI of 271 students was compared at four different time points. The study procedure is visually depicted in Figure 1.

### 3.2. Content Validity

The interpretations of I-CVI for each item (question) and S-CVI/Ave in both questionnaires were acceptable (I-CVI > 0.78, S-CVI > 0.9). Kappa coefficient interpretation was excellent for each question (k* = [0.75–1]). The content validity table for both questionnaires is presented in the Supplementary Materials (Table S1, on page 5).

### 3.3. Calibration

The calibration of examiners was measured at the tooth level. Inter-rater agreement among examiners, as shown by Fleiss’ Kappa, was estimated to be 0.81 for PI, and it was 0.64, 0.89 and 0.91 for Decayed (D/d), Missing (M/m) and Filled (F/f) teeth, respectively. There was a significant level of agreement among examiners (*p* < 0.05). The agreement obtained for PI, M/m and F/f was almost perfect, and a substantial agreement was obtained for D/d between the examiners.

### 3.4. Tests and Analysis

#### 3.4.1. Categorical and Demographic Data

Among all the examined participants, only 32 (10.40%) were caries-free. As the number of 11 year olds was too few (n = 9), they were accounted to the 10-year-old age group category (Table 1).

#### 3.4.2. Plaque Index Measurement of All Three Schools in Time Intervals

The mean PI (SD) pre-intervention for the MSN group was 1.90 (0.65), the MFNO group was 2.02 (0.76) and the C group was 1.96 (0.70). The mean PI in the three groups did not differ significantly from each other at baseline. 

After the oral health education and intervention, in a two-week follow-up, the mean PI were 1.39 in MSN, 1.67 in MFNO and 1.70 in the control group. In all three groups, there was a significant decrease after two weeks of follow-up compared to baseline examination (*p* < 0.01).

In the second follow-up (from two weeks to two months post-intervention), there was an insignificant decrease in MSN and MFNO and an insignificant increase in the C.

In the final follow-up (six-months post-intervention), the mean PI in MSN, MFNO and C were 1.70, 1.69 and 1.92, respectively. From two to six months follow-up, there was a significant increase in mean PI in MSN and C and an insignificant increase in MFNO (Table 2 and Table 3). Additional tables of PI means in school groups and time are attached in the Supplementary Materials (Tables S2 and S3, on page 6).

**Figure 2:**
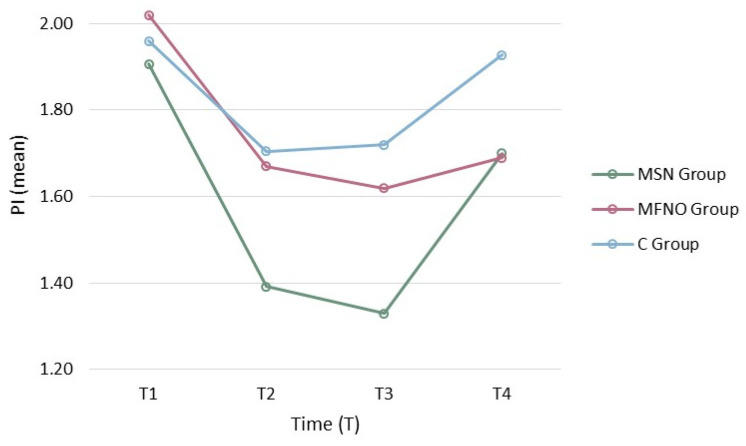
The plaque index mean changes in the study school groups at different time points. MSN: Messages based on Social Norms; MFNO: Messages based on Fear of Negative Outcome; C: Control. Horizontal axis shows “Time (follow-ups)”: T1: Baseline; T2: Two weeks; T3: Two months; T4: Six months. The vertical axis shows “Plaque Index means”.

At six-month follow-up, the PI mean differences in intervention groups (MSN and MFNO) were significant compared to the baseline, as opposed to the C, which was not significant (Figure 2).

#### 3.4.3. Nationality and Dental Filling Effects on PI Changes

Iranian schoolchildren showed a greater reduction in PI mean in the six-month follow-up compared to Afghan schoolchildren, but this difference was not statistically significant (*p* = 0.05). The table and graph of PI changes in nationality groups are presented in the Supplementary Materials (Table S4 and Figure S4, on page 7).

In the six-month follow-up, the PI mean difference between children with no dental fillings and children who already had at least a dental filling (f/F) was statistically significant (*p* < 0.01)*,* and plaque reduction was greater in those who had already had at least a dental filling in their mouth compared to those who had not. The related graph and table are presented in the Supplementary Materials (Table S5 and Figure S5, on page 8).

#### 3.4.4. Nationality and dmft/DMFT Relationship

The mean dmft (SD) for Afghan children was 4.41 (2.01), and for Iranian children it was 3.74 (2.29). The difference was statistically significant (*p* = 0.01). The mean DMFT (SD) for Afghan children was 1.78 (1.25), and for Iranian children it was 1.47 (1.17). No significant difference was found between the DMFT index of Iranian and Afghan children (*p* = 0.05) (Table 4).

#### 3.4.5. Age and dmft/DMFT Relationship

The mean dmfts (SD) for 8-, 9- and 10-year-old children were 4.61 (2.35), 4.47 (1.91) and 2.03 (1.24), respectively. The mean dmft for 10-year-old children was statistically significantly lower than 8- and 9-year-old children (*p* < 0.01). The mean DMFTs for 8-, 9- and 10-year-old children were 1.36 (1.09), 1.64 (1.17) and 2.79 (1.36), respectively. The mean DMFT for 10-year-old children was statistically significantly higher than 8- and 9-year-old children; however, the mean differences of both dmft and DMFT indices between 8- and 9-year-old children were not statistically significant (*p* = 0.86, 0.20) (Table 4).

Overall, the mean (SD) dmft and DMFT indices were 4.24 (2.11) and 1.70 (1.24), respectively, and the median dmft and DMFT indices were 5 and 2, respectively, for all the participants (n = 309). The table of dmft/DMFT indices in school groups is presented in the Supplementary Materials (Table S6, on page 9).

## 4. Discussion

Due to the ease of accessibility, the present study targeted school-going children. The population of public schools is often characterized by many individuals of almost similar age and socioeconomic status. 

Motivating children to improve oral self-care is more cost-effective than constantly running programs to look after them. Having a good understanding of proper oral hygiene practices is crucial for maintaining optimal oral health. Implementing school-based oral health education can greatly benefit a large number of children by improving their knowledge and behavior, all at a minimal cost [41]. The present study showed that nudging primary school children to better oral self-care resulted in better plaque control than traditional, widely used oral health instruction methods.

Children in the third grade were selected for the current study. Establishing healthy oral hygiene habits in childhood would lay the foundation for good dental health in adolescence. When children practice good oral hygiene from an early age, it might remain as a value and potentially make it an enduring habit for them [42]. In addition, at the age of nine, children are in the middle of their mixed dentition, resulting in an appropriate time for evaluating caries experiences in both primary and permanent dentition [43].

Despite Nudge theory’s widespread usage in economics, public policy and healthcare, its application in oral health and dentistry is notably understudied. Although Nudge theory has been shown to be beneficial in promoting healthy behaviors and improving patient outcomes in areas such as smoking cessation and medication adherence, its potential in oral health remains largely unexplored [27]. This lack of research extends to immigrant and refugee populations, with no prior studies focusing on nudge messages for promoting their oral health. Although direct comparisons to other studies were difficult, the authors attempted to analyze the findings alongside similar studies to offer insights and contextualize our results within the existing literature.

In the present study, interventions were designed using two messages from Nudge theory: the effect of social norms and the fear of negative outcome.

First, based on the findings of behavioral economics, one of the strategies for behavior change is to use messages based on social norms. People in society are strongly influenced by the question of what other people will do in a similar situation. Based on this approach, social norms seem to have a great impact on changing people’s habits and behavior. The findings of behavioral economics indicate that statistically people are likely to behave as what they believe to be the prevalent comportment. Moreover, Attitudes that become social norms spread more rapidly in society [44]. A number of recent experiments have examined the impacts of social norms to encourage desired behavior [24,25].

Second, according to the findings of behavioral economics, another way to influence people’s comportment is to show the positive and negative consequences of adopting a behavior. In particular, showing the negative results of behavior to people causes a sense of danger that can lead to a change in a person’s behavior. On average, loss aversion is a greater motivator than the pleasure of gain [26,45,46]. However, a recent review demonstrates that there are some situations where loss aversion might not emerge, and individuals give equal weight to gains and losses [47].

Prior research has indicated the small to moderate impact of nudge interventions on individuals’ food choices and nutritional decision-making [48,49]. Arno et al. [50] conducted a meta-analysis which found that nudge interventions, on average, result in a 15.3% increase in healthier dietary or nutritional decisions. This increase was measured by changes in the frequency of healthy choices or overall calorie intake.

The effectiveness of nutritional nudge methods seems to be influenced by the socioeconomic status of the target population and the delivery mode of nudge. The application of social modeling during group meeting sessions successfully altered patients’ dietary habits and physical activity levels; however, the use of digital devices’ notifications to encourage patients to eat less proved to be unsuccessful. Harbers et al. [48] observed compelling evidence indicating that nudges had a greater impact on individuals from low socioeconomic status groups. On the other hand, Ruggeri et al. [51] found that nudge treatments had a more substantial impact on those with higher socioeconomic levels. This finding is further emphasized by Eze et al. [52], who highlighted the pronounced effects of these interventions on this demographic group.

In the present study, after receiving the oral health package and education, PI was significantly decreased in all three groups in the two-week and two-month follow-ups. These findings were found to be in accordance with the studies conducted by Ingale et al. [43], Shahapur et al. [53] and Zarabadipour et al. [54], where a significant reduction in mean PI was reported after educational intervention. In contrast, Palenstein et al. [55] found no significant reduction in PI after oral health intervention.

In the six-month follow-up, the PI in the C group nearly reverted to pre-study levels, confirming Sharma et al.’s [56] finding that intervention groups showed PI improvements while controls deteriorated. Similarly, Ivanovic et al. [57] found that short-term health education only transiently improved gingival health in schoolchildren, suggesting sustained benefits required prolonged, professional instruction. In the present study, the C group showed a short-term effect, which was in agreement with the findings of Ivanovic et al. [57].

The present study, alongside Evans et al. [58], explored how different communication strategies affect oral hygiene behavior. While Evans et al. highlighted the benefits of positive messaging, we found that fear-based messages were more effective in the long run (six-month follow-up). Both studies reported declining improvements over time, emphasizing the need for ongoing message reinforcement. Our findings support Evans et al.’s observation of the short-term effect of positive messages, particularly those rooted in social norms. There is, however, only limited research comparing fear and positive appeals on oral health, which indicates a notable gap in understanding in this regard [58].

Our results showed that age had a direct relationship with caries experience in permanent dentition and an inverse relationship with caries experience in primary dentition. This finding was in agreement with Soltani et al.’s meta-analysis of caries prevalence among Iranian children [59]. In our study, the reported caries experience was higher than Soltani et al.’s report of average caries experience among Iranian children (overall mean of 3.80 and 2.13 for dmft and DMFT, respectively) [59]. Considering previous research on caries experience in Mashhad school children, our findings revealed that children with an immigrant background had a higher caries experience than those with an Iranian background [60].

Participants with filled teeth in the mouth (f, F) or Iranian nationality showed a greater reduction in PI following the oral health intervention. The existence of restored teeth and Iranian citizenship might suggest enhanced accessibility to dental care facilities and may mirror a more affluent socioeconomic position among parents [61]. This finding aligns with the hypothesis that conversion factors such as parental socioeconomic status, mental health and parental education level might play a crucial role in the translation of health literacy to health outcomes, meaning individuals with favorable social and personal factors (those already privileged) might derive greater health benefits from interventions aimed at enhancing health literacy [62]. Nevertheless, it is crucial to acknowledge the presence of numerous confounding variables at play and methodological limitations, which limit the confidence of any conclusions drawn in this context.

By combining verbal instruction with visual aids, such as leaflets and educational videos, the learning process is enhanced, and a lasting impact might be ensured. The educational content of our study was effectively delivered through a variety of visual aids, including videos and leaflets. Consistent with our finding, Aljafari et al. [63] reported a significant increase in children’s awareness via audio–visual educational interventions for children aged 4 to 10 with a high caries risk. There was only one exposure to the video in this study, unlike those in Lees and Rock’s study [64], where the children took the video home. Repeated exposures could have further led to improvements in oral hygiene. Also, Ghaffari et al.’s findings [8] demonstrated that diverse health education interventions effectively enhance individuals’ knowledge and promote essential self-care behaviors such as brushing and flossing. Our study consistently revealed that all interventions resulted in a reduction of PI in school children.

### 4.1. Strengths of the Study

To the best of the authors’ knowledge, the present study was the first to use the Nudge theory to promote oral self-care among children with immigrant and refugee backgrounds. Moreover, it provided the first report on both the prevalence of caries and the effectiveness of oral health promotion interventions for this population in Iran. These distinctive contributions highlighted how important our research was for informing the next public health programs in Mashhad, Iran.

### 4.2. Limitation

Limitations of this study include the limited representativeness of the selected public schools, which mainly catered to immigrant and refugee children in Mashhad, potentially limiting the generalizability of our findings. Uncontrolled variables such as participants’ personal characteristics, socioeconomic status and prior oral health education from other sources could have caused heterogeneity, potentially confounding the results. A notable limitation of this study was the focus solely on male schoolchildren, which introduces a gender bias and limits the exploration of sex differences in oral hygiene behaviors and outcomes, thus affecting the generalizability of the results. Furthermore, a larger sample size and longer follow-up periods could have strengthened the study’s findings and provided a more comprehensive understanding of the effectiveness and sustainability of the interventions.

### 4.3. Recommendations for Further Studies

Future research may evaluate the comparative effectiveness of various nudge and non-nudge approaches across various social contexts to improve oral health. In addition to providing valuable insights into the most effective strategies for promoting oral health behaviors among different demographic groups, such research may also facilitate the development of customized interventions. The development of innovative approaches and studies aimed at motivating children to take better oral care within various social settings is a pressing need.

## 5. Conclusions

Our findings showed that applying the Nudge theory along with oral health education was more effective than the traditional method of oral health education, and oral health education via visual aids (leaflets and videos) was effective in plaque control.

It was noteworthy that the short-term and long-term effectiveness of the intervention strategies differed. Messages based on social norms demonstrated immediate reductions in PI after two weeks and two months, while messages based on fear of negative outcome showed more sustained effects after six months.

## Figures and Tables

**Table 1:** Demographic data on categorical scale and caries-free individuals.

Category	Subcategory	Number (%)
Nationality	IR ^1^	81 (26.20%)
	AF ^2^	228 (73.80%)
	Total	309 (100%)
Age	8	72 (23.30%)
	9	203 (65.70%)
	10	34 (11%)
	Total	309 (100%)
Caries free	MSN ^3^	11 (3.60%)
	MFNO ^4^	12 (3.90%)
	C ^5^	9 (2.90%)
	Total	32 (10.40%)

^1^ IR: Iranians; ^2^ AF: Afghans; ^3^ MSN: Messages based on “Social Norms”; ^4^ MFNO: Messages based on “Fear of Negative Outcome”; ^5^ C: Control.

**Table 2:** Intragroup comparison of plaque index in different time intervals.

Groups	N	Time Points	PI	Time Intervals	*p*-Value
MSN ^1^	92	baseline (T1)	1.90 (0.65)	T1 to T2	<0.01 *
		two weeks (T2)	1.39 (0.78)	T2 to T3	0.73
		two months (T3)	1.33 (0.77)	T3 to T4	<0.01 *
		six months (T4)	1.70 (0.72)	T4 to T1	<0.01 *
MFNO ^2^	94	baseline (T1)	2.02 (0.76)	T1 to T2	<0.01 *
		two weeks (T2)	1.67 (0.80)	T2 to T3	1.00
		two months (T3)	1.61 (0.77)	T3 to T4	0.90
		six months (T4)	1.69 (0.81)	T4 to T1	<0.01 *
C ^3^	85	baseline (T1)	1.96 (0.70)	T1 to T2	<0.01 *
		two weeks (T2)	1.70 (0.70)	T2 to T3	1.00
		two months (T3)	1.72 (0.78)	T3 to T4	<0.01 *
		six months (T4)	1.92 (0.72)	T4 to T1	1.00
Total	271				

^1^ MSN: Messages based on “Social Norms”; ^2^ MFNO: Messages based on “Fear of Negative Outcome”; ^3^ C: Control; N: Number; PI: Plaque Index (*p*-value *: Statistically significant); Data presented as mean (SD) unless otherwise specified.

**Table 3:** Intergroup comparison of plaque index at different time points.

Time Points	Total PI	Groups	*p*-Value
baseline (T1)	1.96 (0.70)	MSN ^1^ and MFNO	0.82
		MFNO ^2^ and C	1.00
		C ^3^ and MSN	1.00
two weeks (T2)	1.58 (0.77)	MSN and MFNO	0.04 *
		MFNO and C	1.00
		C and MSN	0.02 *
two months (T3)	1.55 (0.79)	MSN and MFNO	0.03 *
		MFNO and C	1.00
		C and MSN	<0.01 *
six months (T4)	1.76 (0.76)	MSN and MFNO	1.00
		MFNO and C	0.11
		C and MSN	0.14

^1^ MSN: Messages based on “Social Norms”; ^2^ MFNO: Messages based on “Fear of Negative Outcome”; ^3^ C: Control; PI: Plaque Index (*p*-value *: Statistically significant); Data presented as mean (SD) unless otherwise specified.

**Table 4:** Comparison of dmft and DMFT indices based on “Nationality” and “Age”.

Nationality	dt ^3^	mt ^4^	ft ^5^	dmft ^1^	*p*-Value
IR^9^	3.23 (2.19)	0.22 (0.52)	0.28 (0.71)	3.74 (2.29)	0.01*
AF ^10^	4.05 (1.88)	0.24 (0.64)	0.12 (0.40)	4.41 (2.01)	
	DT ^6^	MT ^7^	FT ^8^	DMFT ^2^	*p*-value
IR	1.20 (1.05)	0.09 (0.28)	0.19 (0.39)	1.47 (1.17)	0.05
AF	1.63 (1.14)	0.12 (0.35)	0.04 (0.18)	1.78 (1.25)	
Age	dt	mt	ft	dmft	*p*-value
8	4.11 (2.21)	0.35 (0.73)	0.15 (0.49)	4.61 (2.35)	<0.01 *
9	4.05 (1.86)	0.23 (0.60)	0.19 (0.54)	4.47 (1.91)	
10	1.97 (1.24)	0.03 (0.17)	0.03 (0.17)	2.03 (1.24)	
	DT	MT	FT	DMFT	*p*-value
8	1.24 (0.99)	0.11 (0.36)	0.01 (0.12)	1.36 (1.09)	<0.01 *
9	1.44 (1.07)	0.10 (0.32)	0.09 (0.29)	1.64 (1.17)	
10	2.56 (1.28)	0.15 (0.36)	0.09 (0.29)	2.79 (1.36)	

^1^ dmft: caries experience in the primary dentition; ^2^ DMFT: caries experience in the permanent dentition; ^3^ dt—decayed teeth in the primary dentition; ^4^ mt—missing teeth in the primary dentition; ^5^ ft—filled teeth in the primary dentition; ^6^ DT—decayed teeth in the permanent dentition; ^7^ MT—missing teeth in the permanent dentition; ^8^ FT—filled teeth in the permanent dentition; ^9^ IR: Iranians; ^10^ AF: Afghans. (*p*-value *: Statistically significant); Data presented as mean (SD) unless otherwise specified.

## Data Availability

Raw data that support the findings of this study is available from the corresponding author, upon reasonable request.

## Supplementary Materials

The following supporting information can be downloaded at: https://www.mdpi.com/article/10.3390/dj12070228/s1, Figure S1: Effect of social norm (MSN) group reminder, Figure S2: Fear of negative outcome (MFNO) group reminder, Figure S3: Leaflet for the control (C) group that includes only oral health instruction, Figure S4: Nationality and PI changes, Figure S5: Dental filling and PI changes; Table S1: Content Validity of the questionnaires, Table S2: Estimated Marginal Means of “Plaque Index” in school groups, Table S3: Estimated Marginal Means of “Plaque Index” in Time, Table S4: Nationality and PI, Table S5: Dental filling and PI, Table S6: dmft/DMFT comparison in school groups.
