# Supplementary material for: Evaluating the Effectiveness of the Nudge Theory in Improving the Oral Self-Care of Schoolchildren with Refugee and Immigrant Backgrounds in Mashhad, Iran
Source: Dent J (Basel). 2024 Jul 19;12(7):228. doi: 10.3390/dj12070228 (PMC11276547; doi:10.3390/dj12070228)
Supplement: Supplementary file 1 [file dentistry-12-00228-s001.zip › dentistry-3028799-supplementary.pdf]

Supplementary files for "Evaluating the Effectiveness of the Nudge Theory in Improving the Oral Self-Care of Schoolchildren with Refugee and Immigrant Backgrounds in Mashhad, Iran "

CONTENTS

Leaflets designed as reminder for intervention group 1 (MSN)**Error! Bookmark not defined.**

Leaflets designed as reminder for intervention group 2 (MFNO) 3

Leaflets designed as reminder for control group (C)... 4

Table of results... 5

Figure S1. Effect of social norm (MSN) group reminder

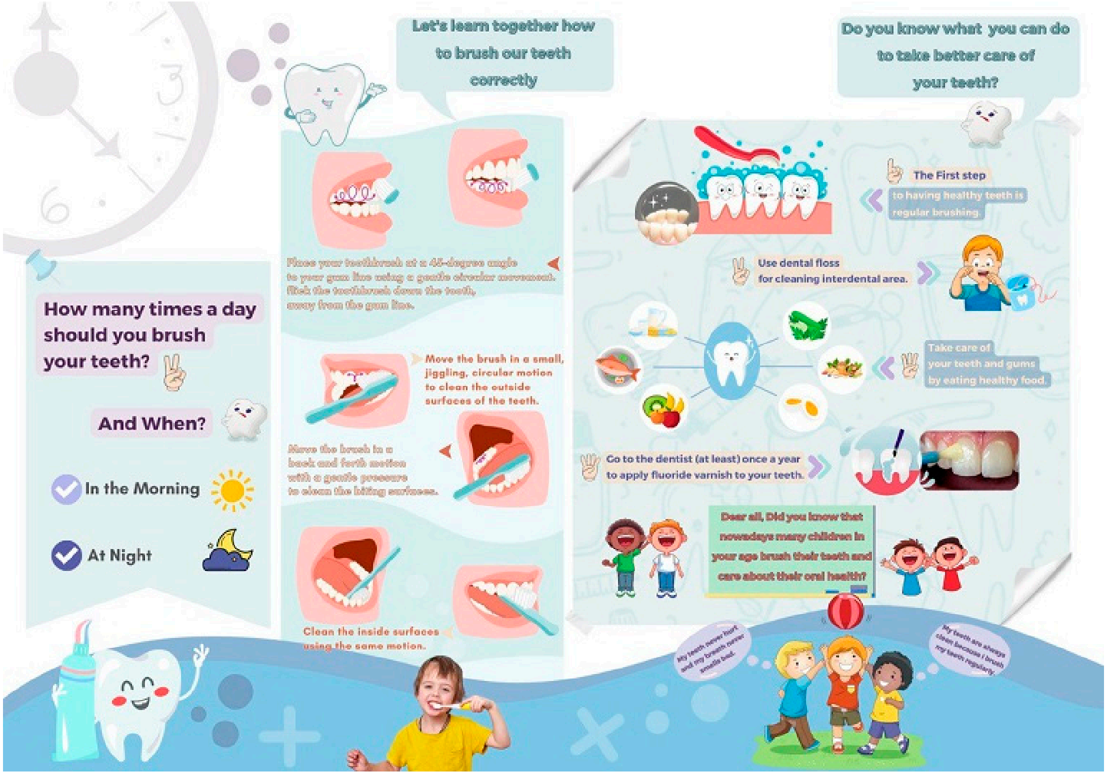

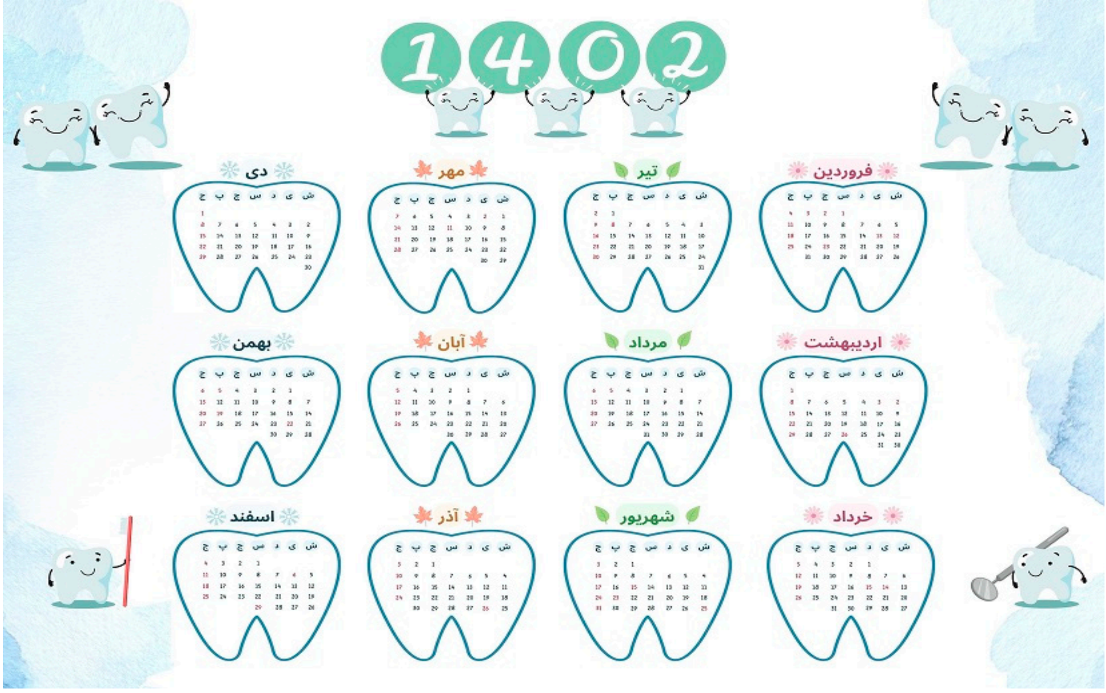

Figure S2. Fear of negative outcome (MFNO) group reminder

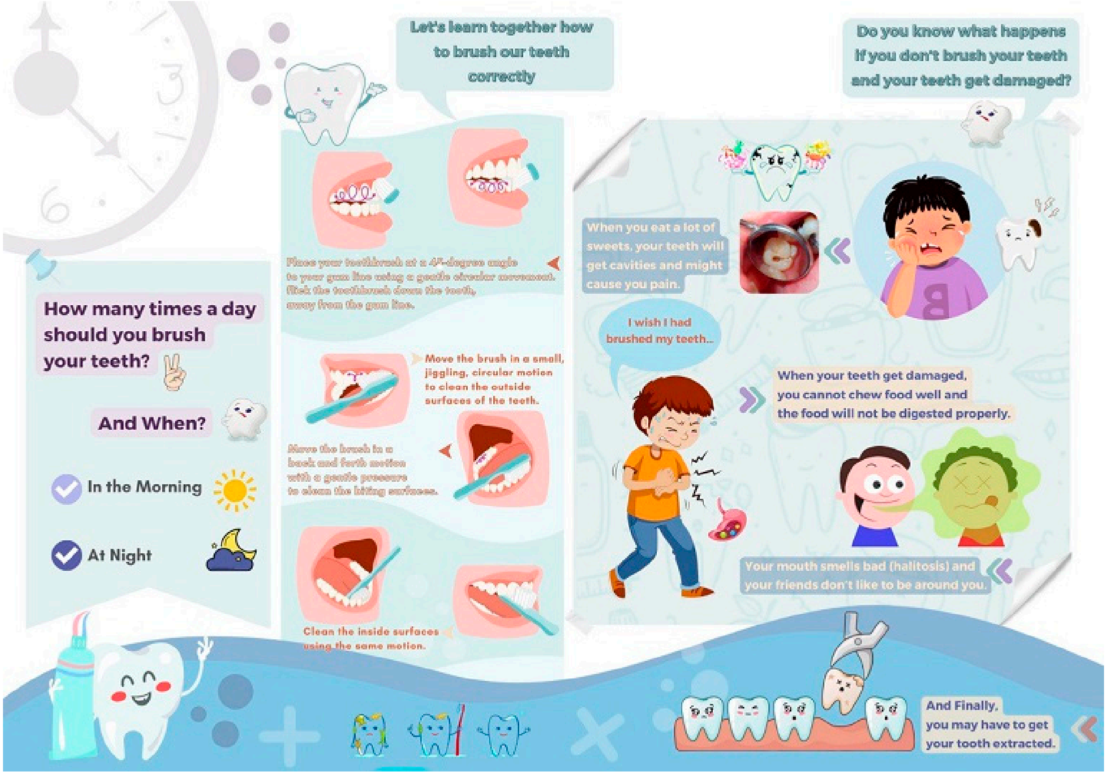

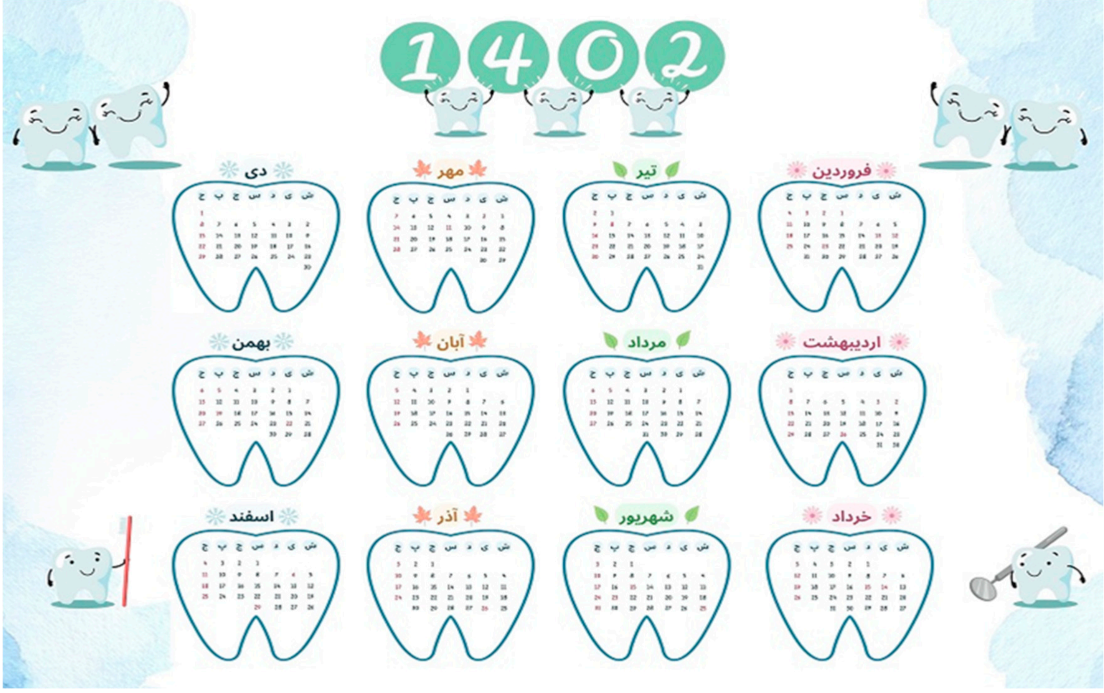

Figure S3. Leaflet for the control (C) group that includes only oral health instruction

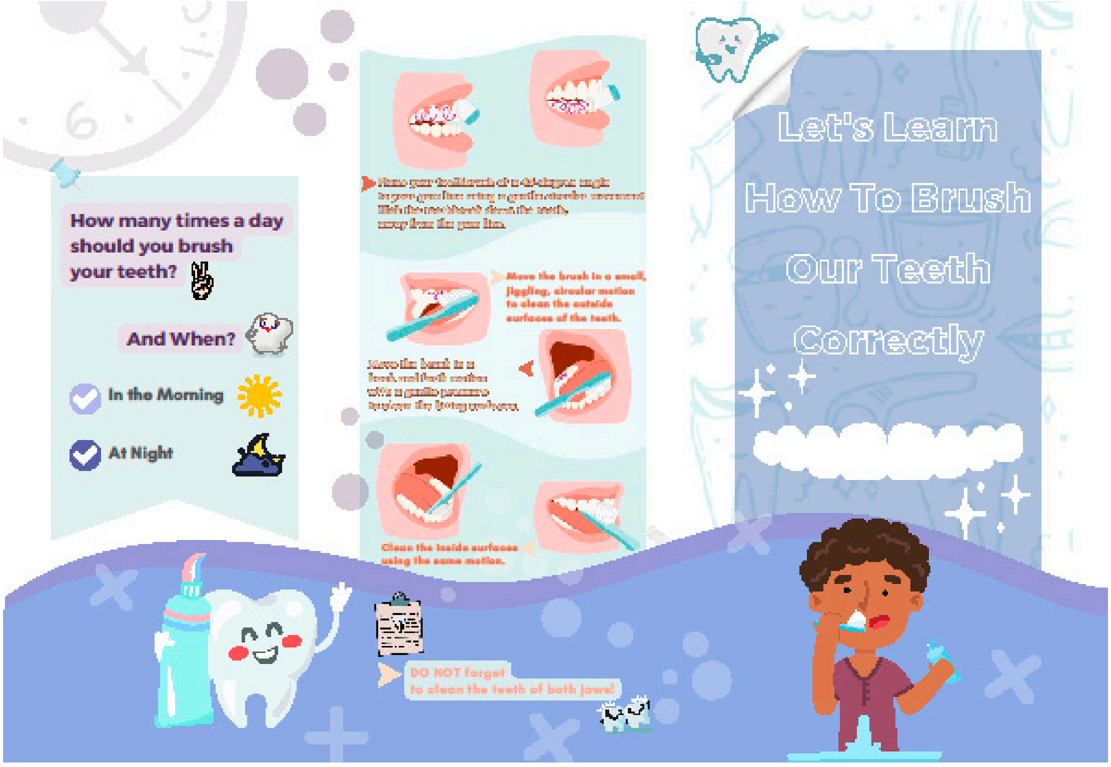

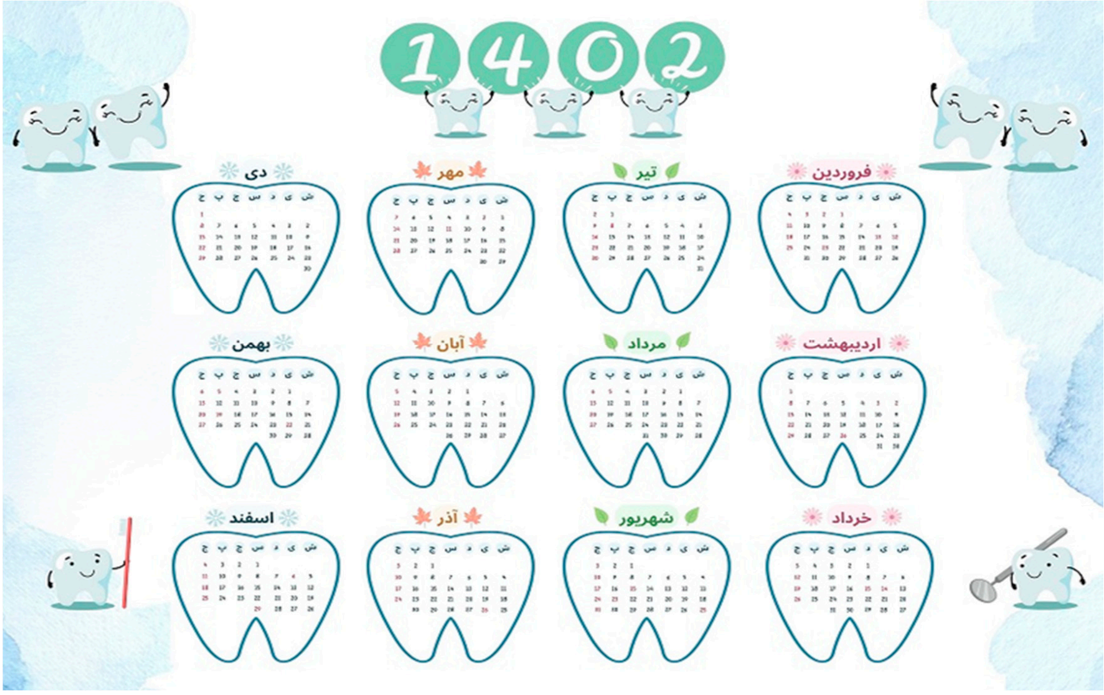

Table S1. Content Validity of the questionnaires

**Questionnaire 1:** ***MSN***					
Likert-scale questions/ Items	Number of Experts in Agreement	**I-CVI(R)**	PC	**K***	Interpretation
**1**	3	0.9	0.0195	0.8980	Excellent
**2**	4	1	0.0009	1	Excellent
**3**	2	0.8	0.1757	0.7573	Excellent
**S-CVI(R)/Ave**	**0.9**				
**Questionnaire 2:** ***MFNO***					
**1**	2	0.9	0.0195	0.8980	Excellent
**2**	4	1	0.0009	1	Excellent
**3**	4	1	0.0009	1	Excellent
**S-CVI(R)/Ave**	**0.9666**				

MSN: *Messages based on "Social Norms"*, MFNO: *Messages based on "Fear of Negative Outcome"*
I-CVI (R): Content validity index for individual items (relevance item level)
S-CVI (R)/Ave: Scale-Content Validity Index/Average of I-CVIs (relevance level)
PC: probability of chance agreement
***K**: Kappa coefficient*

Table S2. Estimated Marginal Means of "Plaque Index" in school groups

Group	Mean	Std. Error	95% Confidence Interval	
			Lower Bound	Upper Bound
**MSN**	1.58	.071	1.44	1.72
**MFNO**	1.75	.070	1.61	1.88
**C**	1.82	.074	1.68	1.97

MSN: *Messages based on "Social Norms",* MFNO: *Messages based on "Fear of Negative Outcome",* C: *Control*

Table S3. Estimated Marginal Means of "Plaque Index" in Time

Time	Mean	Std. Error	95% Confidence Interval	
			Lower Bound	Upper Bound
**T1**	1.96	.043	1.87	2.04
**T2**	1.58	.047	1.49	1.68
**T3**	1.55	.047	1.46	1.65
**T4**	1.77	.046	1.68	1.86

T1: Baseline, T2: Two weeks, T3: Two months, T4: Six months

Table S4. Nationality and PI

Time	Nationality	PI	N
**T1**	**IR**	1.92 (0.66)	72
	**AF**	1.97 (0.72)	199
**T2**	**IR**	1.44 (0.87)	72
	**AF**	1.63 (0.73)	199
**T3**	**IR**	1.37 (0.84)	72
	**AF**	1.61 (0.76)	199
**T4**	**IR**	1.62 (0.74)	72
	**AF**	1.82 (0.76)	199

IR: *Iranians*, AF: *Afghans*, PI*: Plaque Index,* N: *Number*, *T1: Baseline, T2: Two weeks, T3: Two months, T4: Six months*

Figure S4. Nationality and PI changes

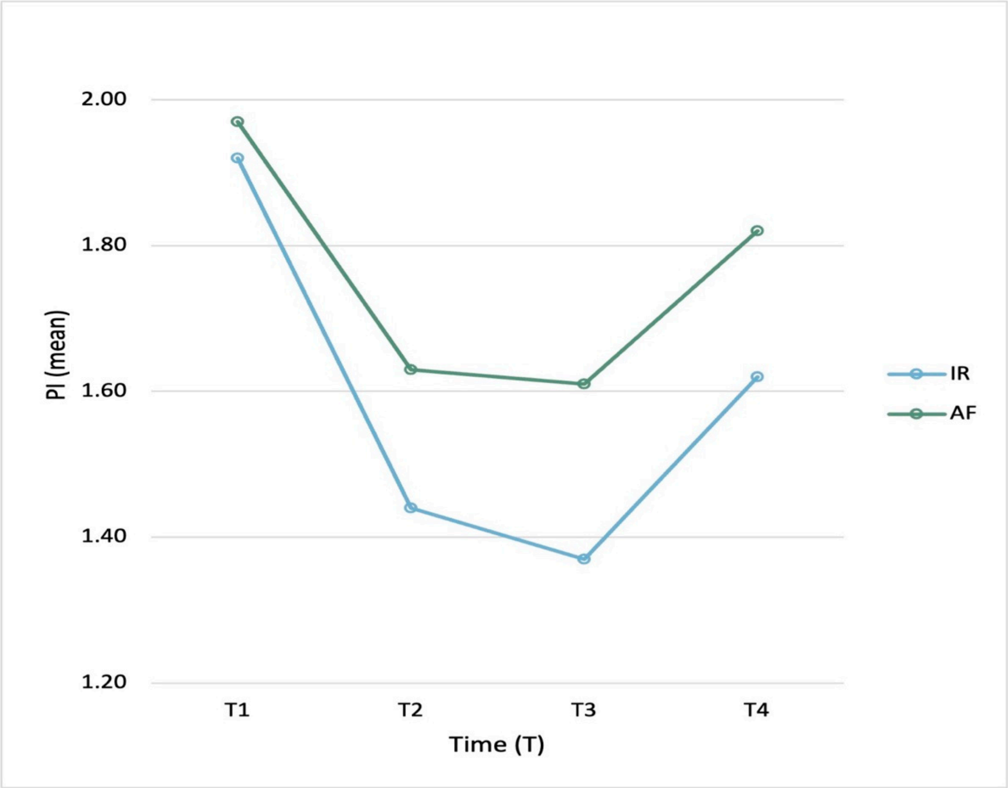

Plaque index means at different time points based on "Nationality"

Horizontal axis shows "*Time (follow-ups)": T**1**: Baseline, T**2**: Two weeks, T**3**: Two months and T**4**: Six months*

Longitudinal axis shows *"PI means"*

IR: Iranians, AF: Afghans

Table S5. Dental filling and PI

Time	F	PI	N
**T1**	**0**	1.96 (0.69)	227
	**1**	1.99 (0.76)	44
**T2**	**0**	1.61 (0.76)	227
	**1**	1.41 (0.83)	44
**T3**	**0**	1.60 (0.78)	227
	**1**	1.30 (0.81)	44
**T4**	**0**	1.83 (0.76)	227
	**1**	1.44 (0.67)	44

F: *Dental Filling*, PI*: Plaque Index,* N: *Number*, *T1: Baseline, T2: Two weeks, T3: Two months, T4: Six months*

Figure S5. Dental filling and PI changes

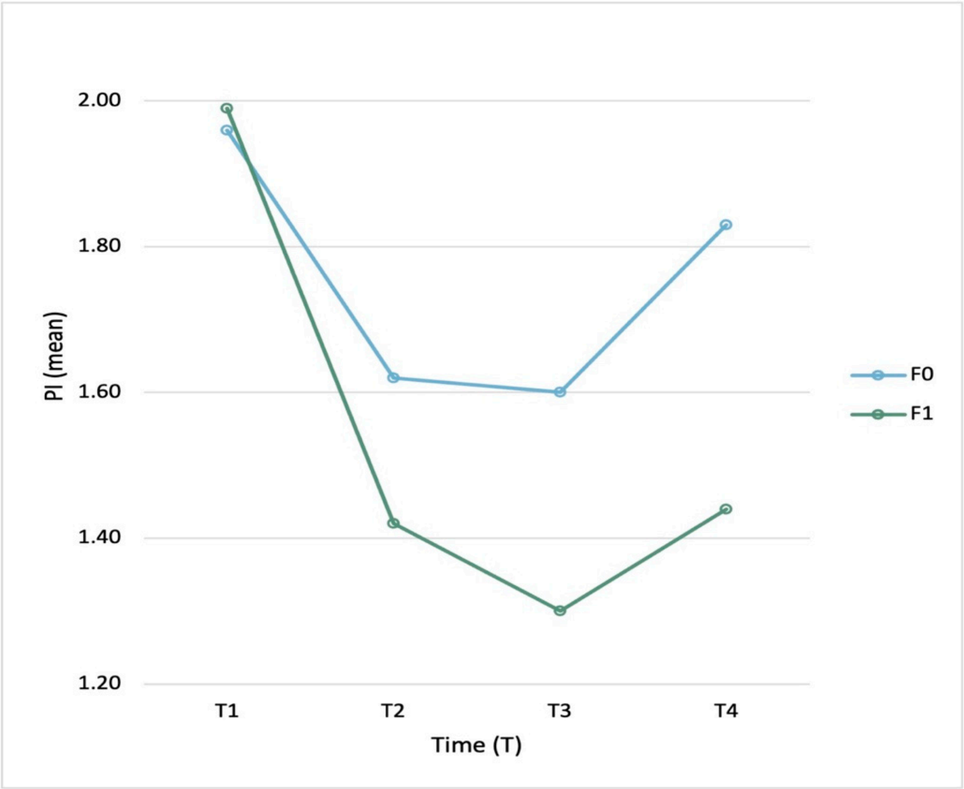

Plaque index means at different time points based on "Dental Filling"

Horizontal axis shows "*Time (follow-ups)*": *T***1***: Baseline, T***2***: Two weeks and T***3***: Two months*

Longitudinal axis shows *"PI means"*

F: dental filling, F0: without fillings, F1: with fillings

Table S6. dmft/DMFT comparison in school groups

Group	N	dt	Mt	Ft	dmft	Median	IQR	P-value
**MSN**	102	3.92 (1.97)	0.19 (0.46)	0.16 (0.44)	4.26 (2.03)	5	1	
**MFNO**	107	3.64 (2.01)	0.20 (0.52)	1.15 (0.55)	3.98 (2.09)	4	2	0.22
**C**	100	3.96 (2.01)	0.33 (0.53)	0.19 (0.53)	4.48 (2.18)	5	3	
Total	309	3.83 (2.00)	0.24 (0.61)	0.17 (0.51)	4.24 (2.10)			
Group	N	Dt	Mt	Ft	DMFT	Median	IQR	P-value
**MSN**	102	1.14 (0.95)	0.10 (0.33)	1.07 (0.25)	1.30 (1.07)	1	2	
**MFNO**	107	1.78 (1.15)	0.12 (0.36)	0.09 (0.29)	1.99 (1.24)	2	2	<0.01 *
**C**	100	1.62 (1.20)	0.11 (0.31)	0.06 (0.24)	1.79 (1.31)	2	2	
Total	309	1.51 (1.14)	0.11 (0.33)	0.07 (0.26)	1.70 (1.24)			

MSN: *Messages based on "Social Norms"*, MFNO: *Messages based on "Fear of Negative Outcome"*, C: *Control*, DMFT: *caries experience in the permanent dentition,* dmft: *caries experience in the primary dentition*, SD*: Standard deviation,* DT – *decayed teeth in the permanent dentition*, MT – *missing teeth in the permanent dentition,* FT – *filled teeth in the permanent dentition*, dt – *decayed teeth in the primary dentition*, mt – *missing teeth in the primary dentition,* ft – *filled teeth in the primary dentition,* IQR*: Interquartile range*

(P-value: Statistically significant), #Data presented as mean (SD) unless otherwise specified.*
